# Inhalation of 5% CO_2_ and activation of ASIC1a: a potential therapeutic approach for Dravet syndrome

**DOI:** 10.1186/s42494-025-00204-8

**Published:** 2025-03-06

**Authors:** Qian Lu, Qi Zhang, Yangyang Wang, Jia Wang, Haiqing Zhao, Qiuhong Wang, Liping Zou

**Affiliations:** 1 Department of Pediatrics, First Hospital of Qinhuangdao, Hebei, 066000 China; 2 Department of Pediatrics, the First Medical Center, Chinese PLA General Hospital, Beijing, 100853 China; 3 Nankai University School of Medicine, Tianjin, 300071 China

**Keywords:** Dravet syndrome, CO_2_, ASIC1a, Seizure, SCN1A

## Abstract

**Background:**

Preferential activation of Acid-sensing ion channel 1a (ASIC1a) by acidosis promotes seizure termination. Studies have found that CO_2_ can reduce neuronal excitability and inhibit seizure activity. Dravet syndrome (DS) is a severe and catastrophic form of epilepsy primarily caused by monoallelic loss-of-function mutations in the *SCN1A* gene. Patients with DS suffer from frequent seizures, which can be triggered by fever and are often resistant to anti-seizure medications. Thus, this study aimed to explore the effect of inhaling 5% CO_2_ and activating ASIC1a against hyperthermia-induced seizures in a mouse model of DS (*Scn1a*^+/-^).

**Methods:**

Mice aged postnatal day 18–28 were divided into four groups: wild type (WT) + air, *Scn1a*^+/-^ + air, WT + CO_2_, and *Scn1a*^+/-^ + CO_2_. Hyperthermia-induced seizures were performed 60 min after gas inhalation. Neuronal damage was assessed using Nissl staining, whereas ASIC1a expression was evaluated through Western blot and immunofluorescence staining.

**Results:**

In the hyperthermia-induced seizure tests, no seizures occurred in WT mice. All mice in the *Scn1a*^+/-^ + air groups experienced seizures. In the *Scn1a*^+/-^ + CO_2_ group, all but one mouse had seizures. CO_2_ inhalation shortened the duration of seizures in *Scn1a*^+/-^ mice, improved electroencephalogram discharge patterns, and reduced neuronal damage in the hippocampus. The ASIC1a protein was mainly expressed in hippocampal neurons, with minor expression observed in astrocytes. The level of hippocampal ASIC1a increased in the *Scn1a*^+/-^ + CO_2_ mice.

**Conclusions:**

After CO_2_ inhalation, the expression of the ASIC1a protein in the hippocampus increased, the duration of hyperthermia-induced seizures was reduced in *Scn1a*^+/-^ mice, and the damage to hippocampal neurons was alleviated.

**Supplementary Information:**

The online version contains supplementary material available at 10.1186/s42494-025-00204-8.

## Background

Acid-sensing ion channel 1a (ASIC1a) is a transmembrane protein sensitive to changes in extracellular pH and is widely expressed in the central nervous system. Acidosis, which results from prolonged seizures and subsequent lactate production that lowers brain pH, has been found to terminate or prevent seizures by creating an acidic environment that activates ASIC1a [1]. The termination of seizure activity by low pH is dependent on ASIC1a [2]. Studies have shown that the knockdown or inhibition of ASIC1a increases seizure severity, whereas its overexpression leads to a reduction in seizure occurrence [2]. These findings suggest that ASIC1a plays an significant role in the pathophysiology of epilepsy.

Inhalation of CO_2_ is known to influence neuronal activity, synaptic transmission, and plasticity [3]. A previous study reported that 10% CO_2_ gas can suppress spike discharges [4]. Schuchmann S et al. found that hyperthermia-induced seizures in rat pup models can quickly be blocked by 5% CO_2_ [5]. Lowering CO_2_ levels increases neuronal excitability and induces epileptic seizure, while increasing CO_2_ levels decreases neuronal excitability and has a sedative effect [6]. Various studies have explored different concentrations of CO_2_ for epilepsy treatment. In rat models of febrile seizure, 5% CO_2_ was reported to reduce seizures activity [5, 7]. A similar anti-seizure effect was also observed in kainic acid-induced epilepsy rat models [8]. Clinically, CO_2_ is a well-tolerated medical gas that has shown rapid and effective anticonvulsant effects in children with absence epilepsy and non-convulsive status epilepticus [9, 10].

Dravet syndrome (DS) is a rare and severe pediatric encephalopathy, with an estimated incidence of 1 in 15,700–40,000 newborns [11]. Approximately 80% of patients with DS have variants in the *SCN1A* gene, mainly truncated mutations. The *SCN1A* gene encodes the α type I sodium channel (Nav1.1), which is highly expressed in the brain, predominantly in neurons, and plays a crucial role in the generation and propagation of action potentials [12]. DS is characterized by early onset and severe seizures, which can lead to developmental delays and movement disorders [13–15]. In children with refractory epilepsy, DS is one of the most common causes of sudden death [16], with an incidence of about 10% before the age of 10 years [17]. A prominent feature of DS is thermal sensitivity: increased ambient or body temperature can induce seizures. Given the low response rate of DS to current anti-seizure medications, new treatments are warranted. This study aimed to examine whether CO_2_ inhalation could activate ASIC1a to provide therapeutic benefits in DS.

## Methods

### Animals

Embryonic stem cells derived from mice with a 129S6/SvEvTac background were used to delete the first exon of the *Scn1a* gene by homologous recombination to obtain *Scn1a*^*tm1Kea*^ mice. These mice were then crossed with inbred C57BL/6 J mice, resulting in F1 mice offspring, as previously described [18]. The *Scn1a*^*tm1Kea*^ mice with the 129S6/SvEvTac background were provided by Dr. Long-Jun Wu (Department of Cell Biology and Neuroscience, School of Arts and Sciences, Rutgers University, USA). All mice were housed in a specific pathogen-free environment with a controlled temperature of 20–26 °C, a humidity level of 40–60%, and a 12-h light–dark cycle. Feed, water, and bedding were autoclaved, and the mice had free access to food and water. The experimental mice were categorized into the wild type (WT) and *Scn1a*^+/-^ groups by PCR genotype identification (Fig. S1). All procedures were conducted in accordance with the guidelines approved by the Institutional Animal Care and Use Committee of Chinese PLA General Hospital (approval number: 2023–56).

### Procedures

Postnatal day 18–28 mice were divided into four groups: WT + air, *Scn1a*^+/-^ + air, WT + CO_2_, and *Scn1a*^+/-^ + CO_2_. The CO_2_ gas mixture consisted of 5% CO_2_ and 95% O_2_. Mice were placed in a sealed transparent plexiglass container (15 cm × 15 cm × 15 cm) with intake and outlet pores, and 10 L/min gas was injected into the container. All mice could move and breathe freely in the container. The gas inhalation lasted for 60 min, and the hyperthermia-induced seizures were performed.

### Electroencephalography (EEG) measurement

Mice were anesthetized, and EEG electrodes were implanted beneath the skull over the cortex by using a stereotaxic instrument; the recording electrodes were positioned 1.7 mm lateral to the midline and 1.5 mm anterior to bregma, while the reference electrode was positioned 1.2 mm lateral to the midline and 1.5 mm anterior to lambda [19]. Baseline EEG recordings were conducted for 0.5–1 h using AE-2010 (Guangzhou Million Andey Electronics Technology, Guangzhou, China) after electrode implantation. Once the mice fully recovered from anesthesia, hyperthermia-induced seizure experiments were performed, and changes in EEG activity were recorded.

### Hyperthermia-induced seizures

Mice were acclimated to a rectal temperature probe for 5 min before an infrared heat lamp was turned on to gradually elevate their core body temperature by 0.5 °C every 2 min until either a seizure occurred or a temperature of 43 °C was reached. Mice maintained at 43 °C were observed for 3 min to check for seizures and then they were placed on a cold metal surface to cool down to 37 °C. If a seizure occurred during the hyperthermia induction, heating was stopped immediately. Seizure grades were assessed using the classic Racine scale [20]. The temperature of seizure onset, duration of seizure, and grade were recorded. Recording began when visible seizure activity was observed and was halted when the mice stopped seizure with cessation of movement and flaccid weakness in the limbs.

### Western blot (WB)

Total proteins from hippocampal tissue were extracted using the BCA Protein Extraction Kit (C190501, YangGuangBio, Beijing, China). The proteins were separated by electrophoresis and subsequently transferred to a polyvinylidene fluoride membrane (Merk Millipore, Darmstadt, Germany). The membranes were incubated overnight at 4 ℃ with rabbit antibody β-actin (1:10000) and ASIC1a (1:1000). After washing with TBST, the membranes were incubated with a secondary antibody, goat anti-rabbit IgG (1:10,000). Results were visualized and saved, and Image J software (NIH, Bethesda, USA) was utilized for gray-level analysis.

### Immunofluorescence staining

Tissue sections were prepared by embedding in paraffin. Nissl staining was performed on a portion of the sections. Other sections were incubated with primary antibodies ASIC1a (1:100), NeuN (1:500), and GFAP (1:500), followed by incubation with fluorescent secondary antibodies. The sections were then sealed and observed. Neuron counts and ASIC1a immunofluorescence were analyzed in the region of hippocampal CA1, CA3, and dentate gyrus (DG). The intensities of ASIC1a expression were quantified using the mean integrated optical density with Image J.

### Statistical analysis

Normality of the data was assessed using the Kolmogorov–Smirnov normality test. If the parameters were not a normal distribution, the nonparametric Mann–Whitney *U* test was employed; otherwise, Student’s *t*-test was conducted. Multiple samples were compared using ANOVA or the Kruskal–Wallis H test. *P* < 0.05 was considered to be statistically significant. The data were analyzed with SPSS 24.0 (IBM, Armonk, NY, USA) and GraphPad Prism 7 software (GraphPad Software, Boston, MA).

## Results

### After CO_2_ inhalation, the duration of seizures was shortened in *Scn1a*^+/-^ mice

As the temperature increased, the mice began to groom, followed by increased activities and even jumping. No seizures were observed in the WT + air group (*n* = 24) or the WT + CO_2_ group (*n* = 18). All mice in the *Scn1a*^+/-^ + air group (*n* = 27) had seizures. In the *Scn1a*^+/-^ + CO_2_ group (*n* = 18), all but one mouse had a seizure.

**Fig. 1:**
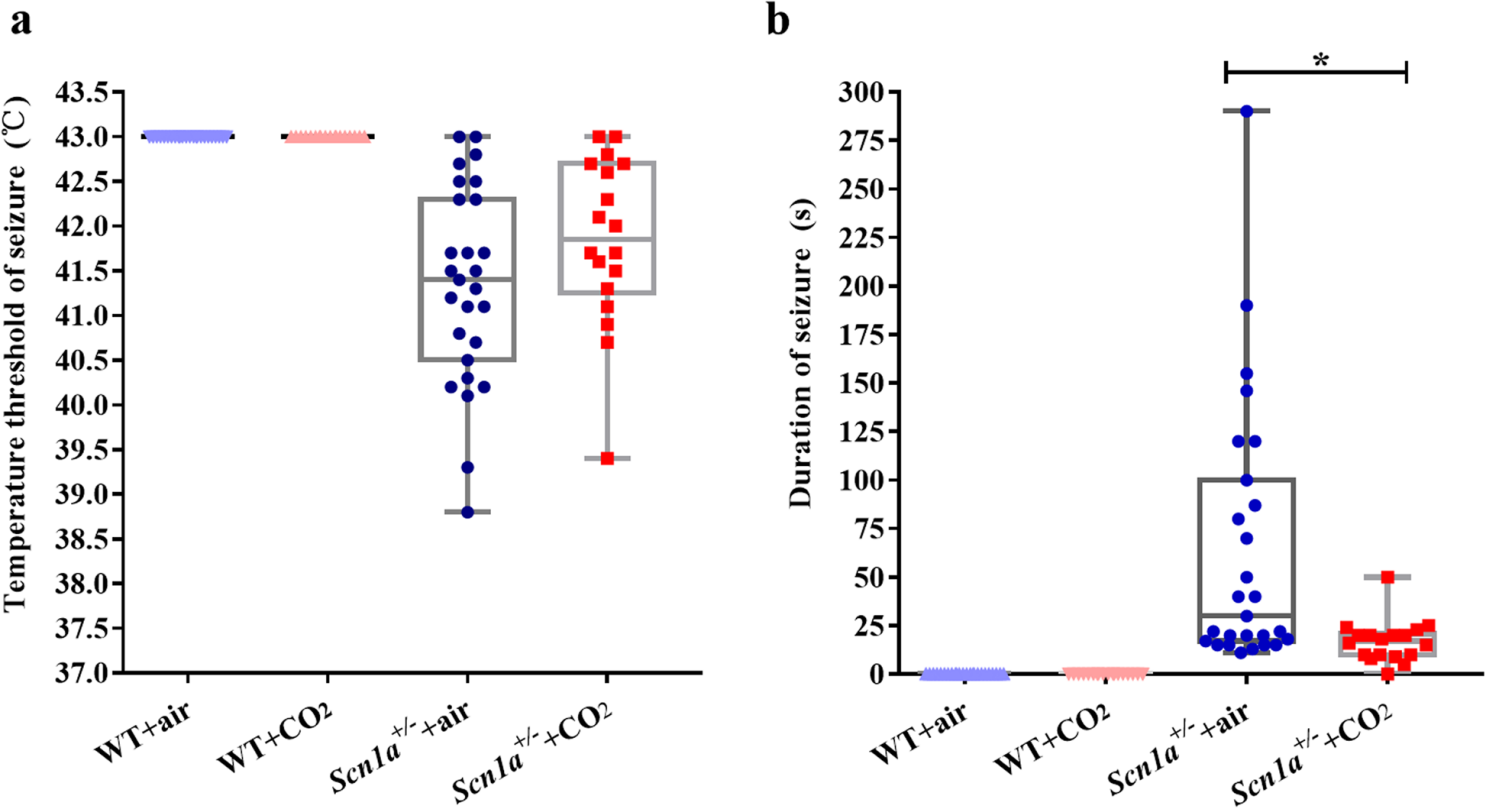
Comparison of seizures in each group of mice. **a** shows the temperature at which convulsions occur, and **b** shows the duration of convulsions. The blue triangle represents the WT + air group, the red triangle represents the WT + CO_2_ group, the blue dot represents the *Scn1a*^+/-^ + air group, and the red square represents the *Scn1a*^+/-^ + CO_2_ group. * indicates *P* < 0.05

A comparison of seizure severity in *Scn1a*^+/-^ group mice with different interventions is shown in Fig. 1. We found no significant difference in the temperature threshold of seizures between the *Scn1a*^+/-^ + air group and the *Scn1a*^+/-^ + CO_2_ group (41.40 [40.50, 42.30] °C vs. 41.70 [41.10, 42.70] °C, *P* = 0.215). However, the duration of seizure in the *Scn1a*^+/-^ + CO_2_ group was significantly lower than that in the *Scn1a*^+/-^ + air group (16.00 [10.00, 20.00] s vs. 35.00 [17.00, 100.00] s, *P* = 0.002). The median score of the Racine scale in different groups of mice was grade V, with no significant difference among them (*P* = 0.076).

### After CO_2_ inhalation, the EEG discharge of *Scn1a*^+/-^ mice was reduced

Since neither the WT + CO_2_ nor the WT + air groups had seizures, they were uniformly classified as the WT group. EEG was performed in the WT group, *Scn1a*^+/-^ + air group, and *Scn1a*^+/-^ + CO_2_ group. At normal body temperature, the EEG of the WT group primarily displayed α and β waves, with no significant abnormalities observed. Even at 43 ℃, no obvious abnormality was observed (Supplementary Figs. S2a–c). In the *Scn1a*^+/-^ + air group, the EEG also showed α and β waves at normal body temperature, but with higher amplitude compared to the WT group, along with occasional sharp and spike waves. As the temperature increased, multiple spikes and sharp waves appeared, accompanied by increased amplitude. During the seizure episodes, the EEG in the *Scn1a*^+/-^ + air group was dominated by high-amplitude sharp and spiked waves (Supplementary Figs. S2d–f). While in the *Scn1a*^+/-^ + CO_2_ group, EEG discharges were less than that in the *Scn1a*^+/-^ + air group as the temperature increased (Supplementary Figs. S2g–i).

### After CO_2_ inhalation, damage to hippocampal neurons was alleviated

In the WT group, neuronal cells in the CA1 region were neatly arranged with clear cell boundaries and abundant Nissl bodies. In the CA3 region, the cells were large and dispersed, while in the DG region, the cells were numerous and closely arranged. In the *Scn1a*^+/-^ + air group, neuronal cells exhibited blurred boundaries, disordered arrangement, and a decrease in Nissl bodies, which signified cell necrosis. After CO_2_ inhalation, the damage of neuronal cells in the hippocampal region of *Scn1a*^+/-^ mice was alleviated.

**Fig. 2:**
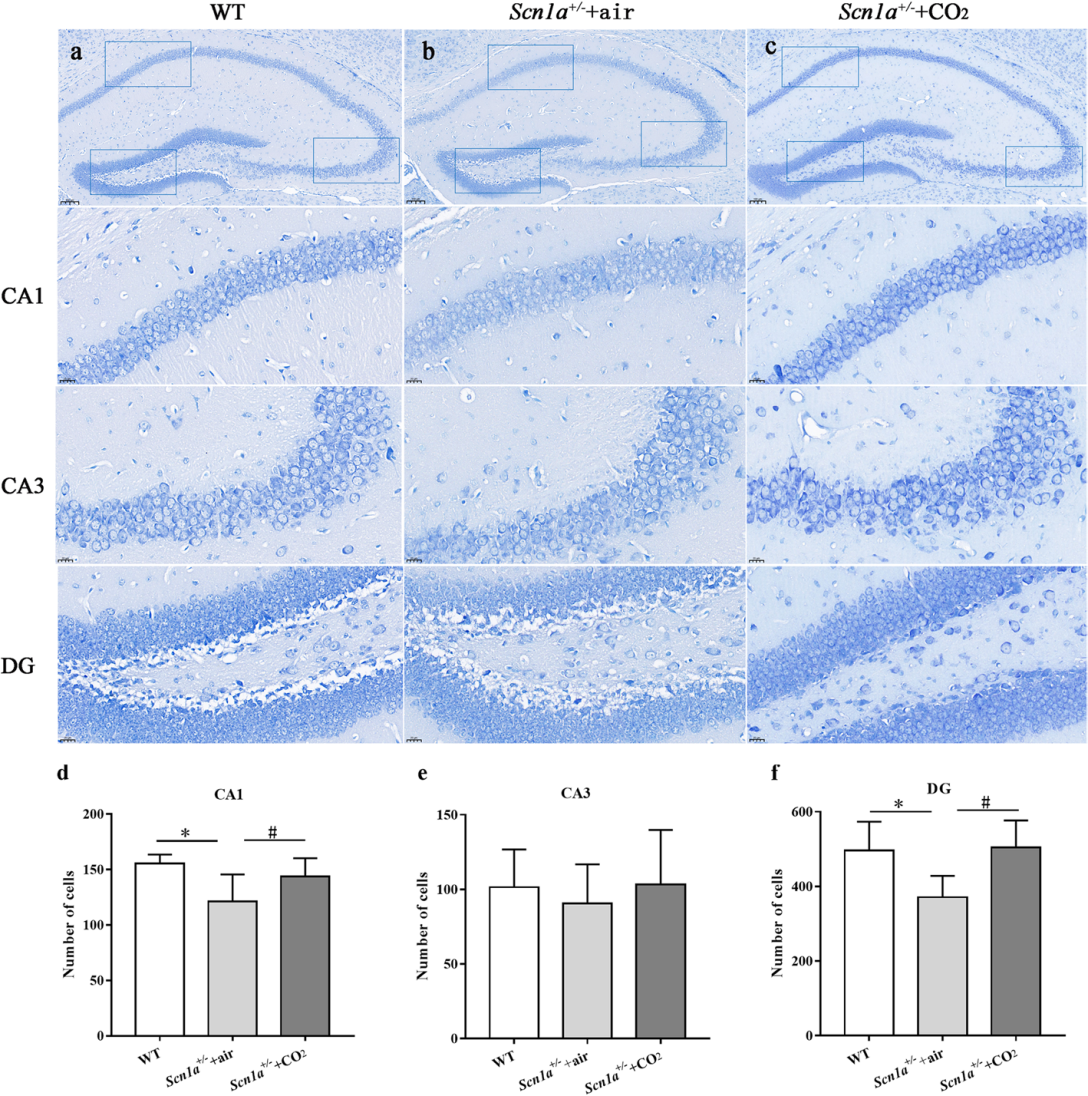
Reduced hippocampal cell damage in *Scn1a*^+/-^ + CO_2_ group. **a**, **b** and **c** Nissl staining in the CA1, CA3 and DG region. **d**, **e**, and **f** show the number of neurons in different regions of the hippocampus. * indicates that *P* < 0.05 was compared between WT group and *Scn1a*^+/-^ + air group. # indicates that *P* < 0.05 was compared between *Scn1a*^+/-^ + air group and *Scn1a*^+/-^ + CO_2_ group

In the CA1 region, the number of neurons in the *Scn1a*^+/-^ + air group was significantly lower than that in the WT group (*P* = 0.001). The neurons in the *Scn1a*^+/-^ + CO_2_ group were similar to those in the WT group (*P* > 0.05) and significantly higher than those in the *Scn1a*^+/-^ + air group (*P* = 0.044). Similar results were observed in the DG region. The number of neurons in the CA3 region in the three groups showed no significant differences (Fig. 2).

### Expression of the ASIC1a protein in the hippocampus

**Fig. 3:**
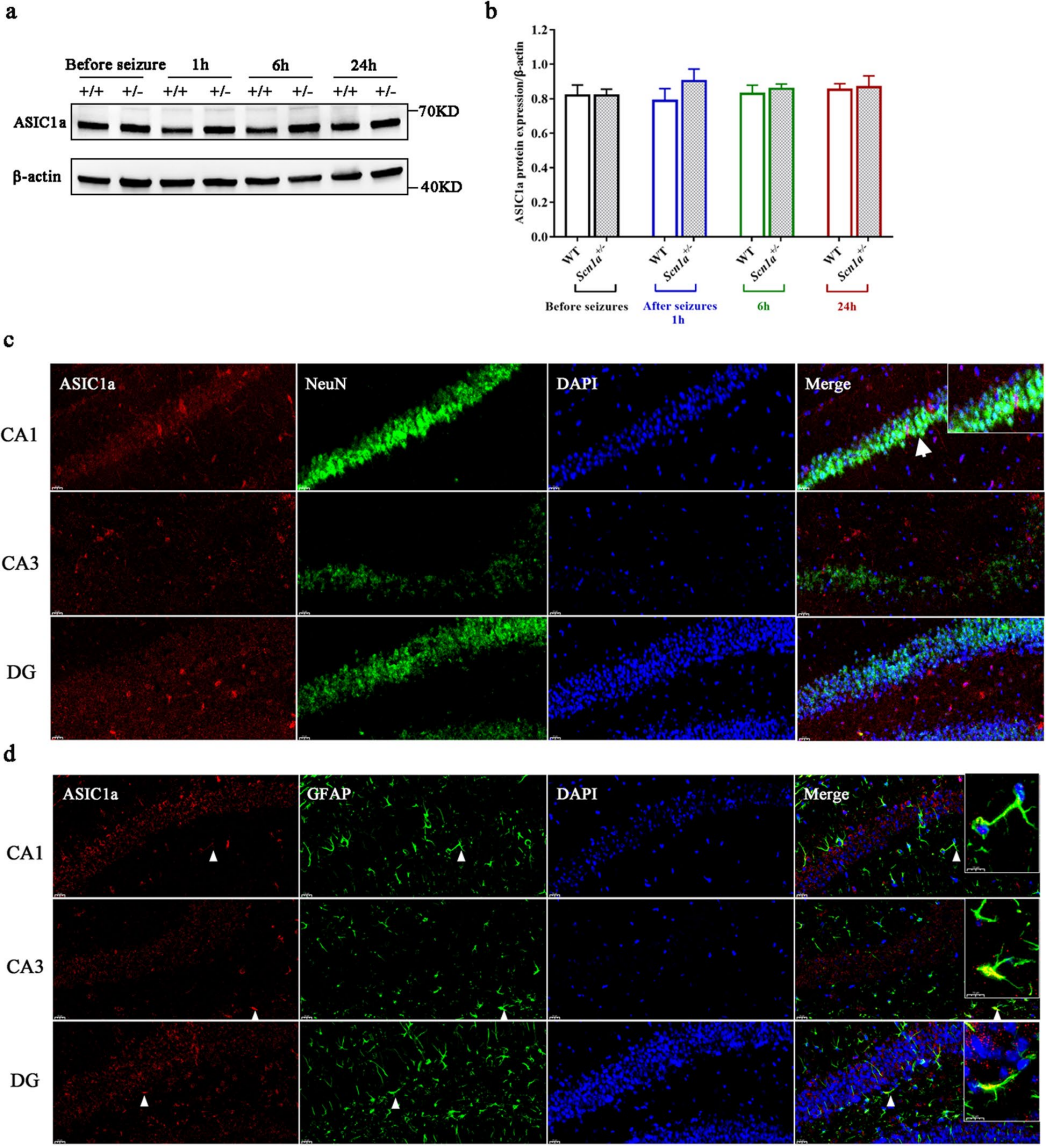
There was no significant difference in the expression of ASIC1a protein between different groups of mice before and after seizures. **a** shows the WB results, + / + is WT mice, and +/- is *Scn1a*^+/-^ mice. **b** shows the relative expression levels of ASIC1a protein/β-actin. Different colors indicate different time points (*n* = 4). **c** shows the expression of ASIC1a protein in neurons. Red indicates the ASIC1a protein, green indicates the neurons, and 4',6-diamidino-2-phenylindole (DAPI) marks the nuclei. The white arrow indicates that the ASIC1a is co-expressed with NeuN. **d** shows that ASIC1a protein is expressed in astrocytes in small amounts. Red indicates the ASIC1a protein, green indicates the astrocytes, and DAPI marks the nuclei. The white triangle indicates that ASIC1a is co-expressed with GFAP, shown in the enlarged image on the upper right in Merge

The expression of the ASIC1a protein before and after hyperthermia-induced seizures was assessed by WB in the hippocampus of WT and *Scn1a*^+/-^ mice. The results showed a basal expression of the ASIC1a protein in the hippocampus of both WT and *Scn1a*^+/-^ mice before seizures, and we found no significant difference in ASIC1a expression between the two groups (*P* = 0.998), indicating that *Scn1a* gene haploinsufficiency did not affect its expression. At 1, 6, and 24 h after hyperthermia, there were no significant difference in ASIC1a protein expression between WT and *Scn1a*^+/-^ mice (*P* = 0.292,* P* = 0.629, and *P* = 0.842, respectively). We also found no significant differences in the expression of ASIC1a in the same group of mice at different time points (*P* = 0.877 and *P* = 0.526, respectively; Fig. 3a and b). These results indicated that neither hyperthermia nor seizure affect the expression of ASIC1a.

The expression of the ASIC1a protein was evaluated by immunofluorescence staining in the hippocampus at 24 h after seizures. The results showed that the expression distribution of ASIC1a in the hippocampus coincided with the expression of NeuN, a protein marker for mature neurons, indicating that the ASIC1a protein was expressed in neurons (Fig. 3c). Some ASIC1a was found co-expressed with the astrocyte marker GFAP, indicating that the ASIC1a protein was also expressed in astrocytes (Fig. 3d).

### After CO_2_ inhalation, the expression of the ASIC1a protein in the hippocampus of *Scn1a*^+/-^ mice increased and the proliferation of astrocytes was reduced

**Fig. 4:**
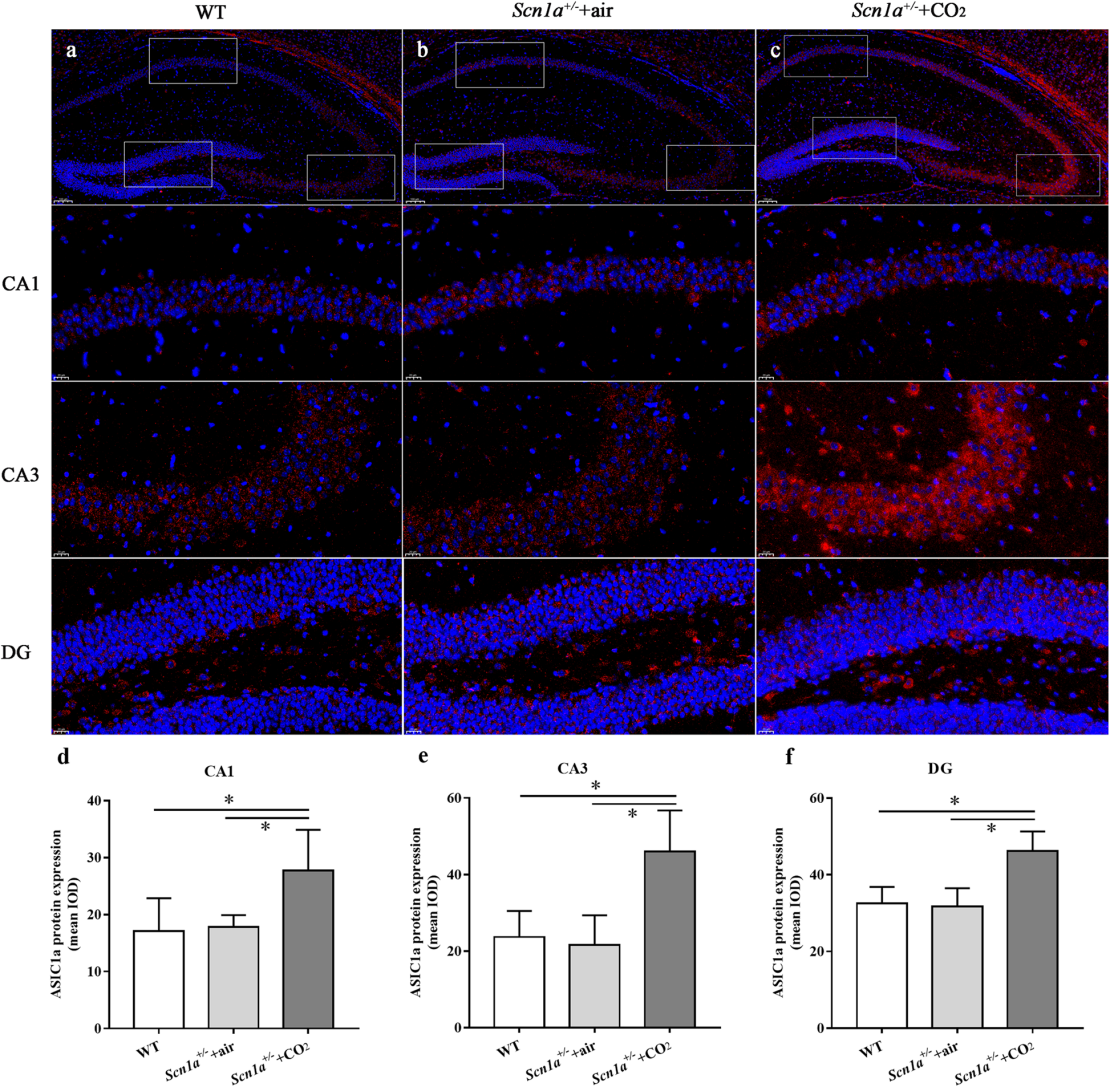
Increased expression of ASIC1a in the hippocampal region of *Scn1a*^+/-^ + CO_2_ group. **a**, **b** and **c** show the expression of ASIC1a in the CA1, CA3 and DG regions. Red indicates the ASIC1a protein, and blue marks the nuclei. **d**, **e**, and **f** show the quantification of mean integral optical density (IOD) from ASIC1a. The expression of ASIC1a in *Scn1a*^+/-^ + CO_2_ group was higher than that in WT and *Scn1a*.^+/-^ + air groups. * indicates *P* < 0.05

The analysis of ASIC1a protein expression in different groups showed that the level of ASIC1a protein in the hippocampus was significantly increased in the *Scn1a*^+/-^ + CO_2_ group compared to both the *Scn1a*^+/-^ + air group and the WT group (Fig. 4 and Fig. S3).

**Fig. 5:**
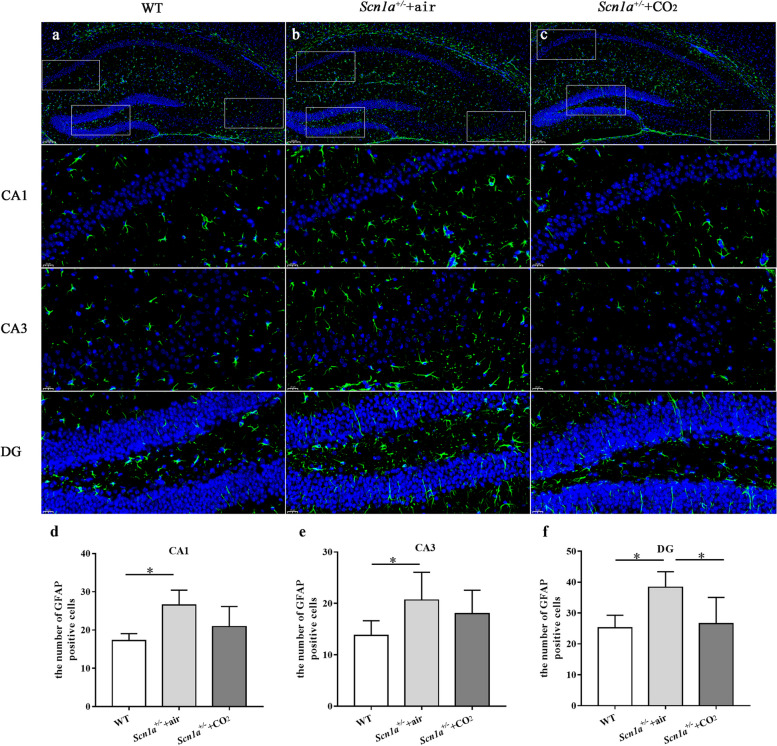
Inhalation of CO_2_ reduced the proliferation of astrocytes in the hippocampus after seizure in the *Scn1a*^+/-^ group. **a**, **b** and **c** show the expression of GFAP in the CA1, CA3 and DG regions. **d**, **e**, and **f** show the number of astrocytes in the hippocampus (*n* = 9). * indicates *P* < 0.05

We also analyzed the hippocampal astrocytes (GFAP-positive cells) in the different groups of mice. Astrocyte proliferation in the CA1 and CA3 region of the *Scn1a*^+/-^ + air group was significantly higher than that in the WT group (*P* < 0.001). After CO_2_ inhalation, the number of astrocytes was roughly equivalent to that in the WT group. In the DG region, astrocyte proliferation in the *Scn1a*^+/-^ + air group was higher than that in the WT (*P* < 0.001) and *Scn1a*^+/-^ + CO_2_ groups (*P* = 0.01). The number of astrocytes in the *Scn1a*^+/-^ + CO_2_ group was similar to that in the WT group (*P* = 0.97; Fig. 5).

## Discussion

All *Scn1a*^+/-^ mice experienced seizures induced by hyperthermia, which provided convenience for studying the relationship among epilepsy of DS, CO_2,_ and ASIC1a. Previous studies, such as those involving kainic acid-induced seizures, indicated that inhalation of CO_2_ can reduce pH levels and seizure activity [8]. This study utilized the *Scn1a*^+/-^ mice model to investigate the effects of 5% CO_2_ on seizures. Remarkably, after 5% CO_2_ inhalation, the duration of seizures was shortened, abnormal discharge was reduced, and neuronal damage in the hippocampus of *Scn1a*^+/-^ mice was alleviated. Further investigation revealed that after 5% CO_2_ inhalation, the expression of the ASIC1a protein in the hippocampus increased, while the proliferation of astrocytes decreased, ultimately providing neuroprotection. These findings expand the therapeutic potential of CO_2_ and suggest a novel treatment approach for patients with DS.

This study found that the baseline expression of the ASIC1a protein in *Scn1a*^+/-^ mice was comparable to that in WT mice, indicating that *Scn1a* gene knockout did not affect the expression of ASIC1a. Contrarily, some studies using Sprague–Dawley rats showed that hyperthermia-induced seizures significantly increased ASIC1a protein expression in a model of complex febrile convulsions [21]. However, in this study, neither hyperthermia nor seizures affected ASIC1a expression in *Scn1a*^+/-^ mice. The discrepancy may arise from differences in the animal models used. Both studies involved hyperthermia-induced seizures, the current study utilized a genetically fever-sensitive *Scn1a* knockout model, whereas the referenced study used normally developing 10-day-old pups.

In this study, mice at 18 days postnatal were selected to start the experiments. First, the expression of Nav1.1 is low in the initial days of postnatal life until the second and third postnatal weeks when it increases significantly [22]. Second, *Scn1a*^+/-^ mice exhibit recurrent seizures and sporadic deaths during postnatal days 21–27 [23, 24]. *Scn1a*^+/-^ mice have been reported to have seizures induced by hyperthermia as early as 18 days after birth [11]. Moreover, previous studies have also chosen to start the intervention experiment around 18 days [25, 26]. Therefore, the age of 18–28 days after birth may be a optimal experimental period.

The role of the ASIC1a protein in epilepsy remains controversial. In pilocarpine-induced epileptic mice, seizures were suppressed following drug-inhibition of the ASIC1a protein, indicating that the ASIC1a protein promotes epilepsy [27]. Conversely, other studies have reported that ASIC1a protein expression was reduced in the cortex of patients with focal cortical dysplasia compared with normal cortex (excluding epilepsy or neurological disorders) [28]. Additionally, mice with ASIC1a deletion had severe seizures in chemically induced epilepsy models, whereas overexpression of the ASIC1a protein significantly reduced the severity of seizures [2]. In the current study, although seizures did not alter the expression of the ASIC1a protein, CO_2_ inhalation increased the expression of the ASIC1a protein and alleviated seizures. Thus, the increase of the ASIC1a protein in DS may play an anti-seizure role, contributing to our understanding of ASIC1a function in DS.

ASIC1a is mainly expressed in neurons, especially GABAergic neurons, and less expressed in glutaminergic neurons and glial cells [29]. Our immunofluorescence results confirmed that the ASIC1a protein is expressed in neurons (NeuN) and also in astrocytes, which was consistent with the existing literature. Notably, ASIC1a is highly expressed in reactive astrocytes in patients with temporal lobe epilepsy [30]. Inhibition of astrocyte ASIC1a expression alleviates spontaneous seizures, wherera restoration of astrocyte ASIC1a expression in ASIC1a knockout mice increases spontaneous seizures, indicating that the involvement of astrocytic ASIC1a in epilepsy [30]. In our study, we found that the number of astrocytes decreased after CO_2_ inhalation, which likely contributed to the reduction in seizure activity. Unfortunately, we did not conduct a quantitatively analysis the ASIC1a expression in astrocytes of *Scn1a*^+*/−*^ mice before and after CO_2_ inhalation.

This study also had several limitations. First, it only focused on hyperthermia-induced seizures and did not assess spontaneous seizures, leaving the effect of CO_2_ inhalation on spontaneous seizures unclear. Second, this study did not conduct experiments aimed at reducing the expression of the ASIC1a protein to prove the relationship among ASIC1a, CO_2_, and DS. Third, while the survival rate of both groups of mice was similar during the 24 h after a heat-induced seizure, unfortunately, we did not extend our observations over a longer period.

## Conclusions

In this study, inhalation of CO_2_ by *Scn1a*^+/-^ mice increased ASIC1a protein expression, reduced the duration of seizures, mitigated hippocampal neuronal damage, and provided neuroprotection. Our findings suggested that inhalation of 5% CO_2_ may offer a promising strategy for the treatment of DS, and activation of the ASIC1a protein representing a novel therapeutic direction for DS.

## Supplementary Information

Supplementary Material 1.

## Data Availability

The data that support the findings of this study are available from the corresponding author upon reasonable request.

## Abbreviations

ASIC1a: Acid-sensing ion channel 1a
DS: Dravet syndrome
EEG: Electroencephalography
WB: Western blot
WT: Wild type
