# Supplementary material for: Inhalation of 5% CO2 and activation of ASIC1a: a potential therapeutic approach for Dravet syndrome
Source: Acta Epileptol. 2025 Mar 6;7:19. doi: 10.1186/s42494-025-00204-8 (PMC11960217; doi:10.1186/s42494-025-00204-8)
Supplement: Supplementary file 1 — Supplementary Material 1. [file 42494_2025_204_MOESM1_ESM.docx]

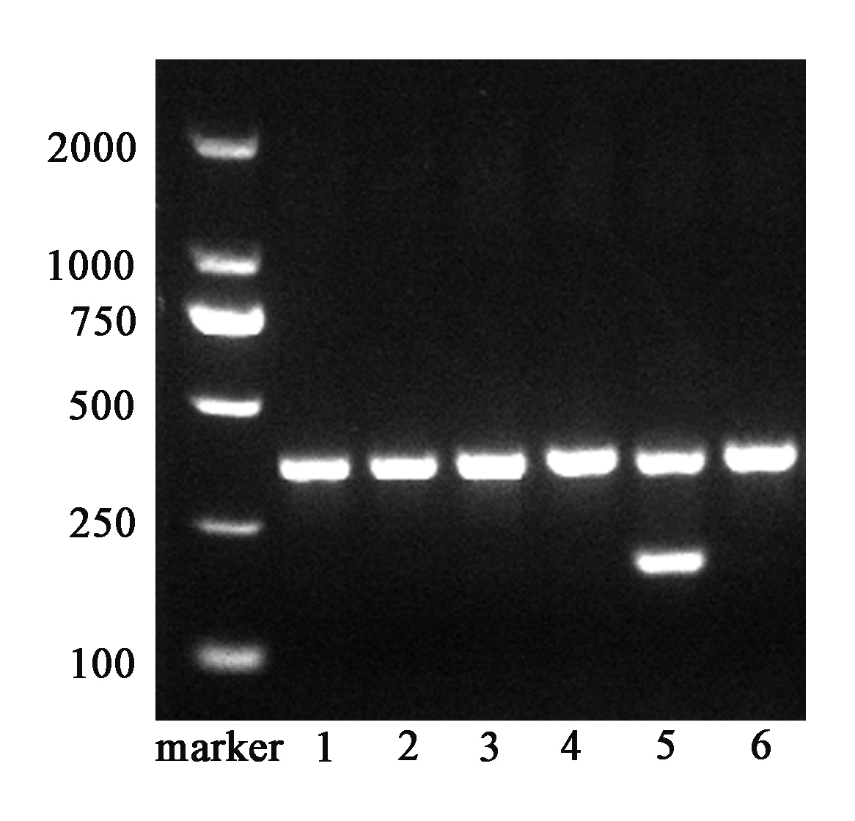


**Figure S1:** The PCR product of WT mice was 280bp, and that of *Scn1a^+/-^* mice was 150bp. 1-6 represented the 6 mice for genotype identification, mouse 5 was *Scn1a^+/-^* mouse, and the rest were WT mice.


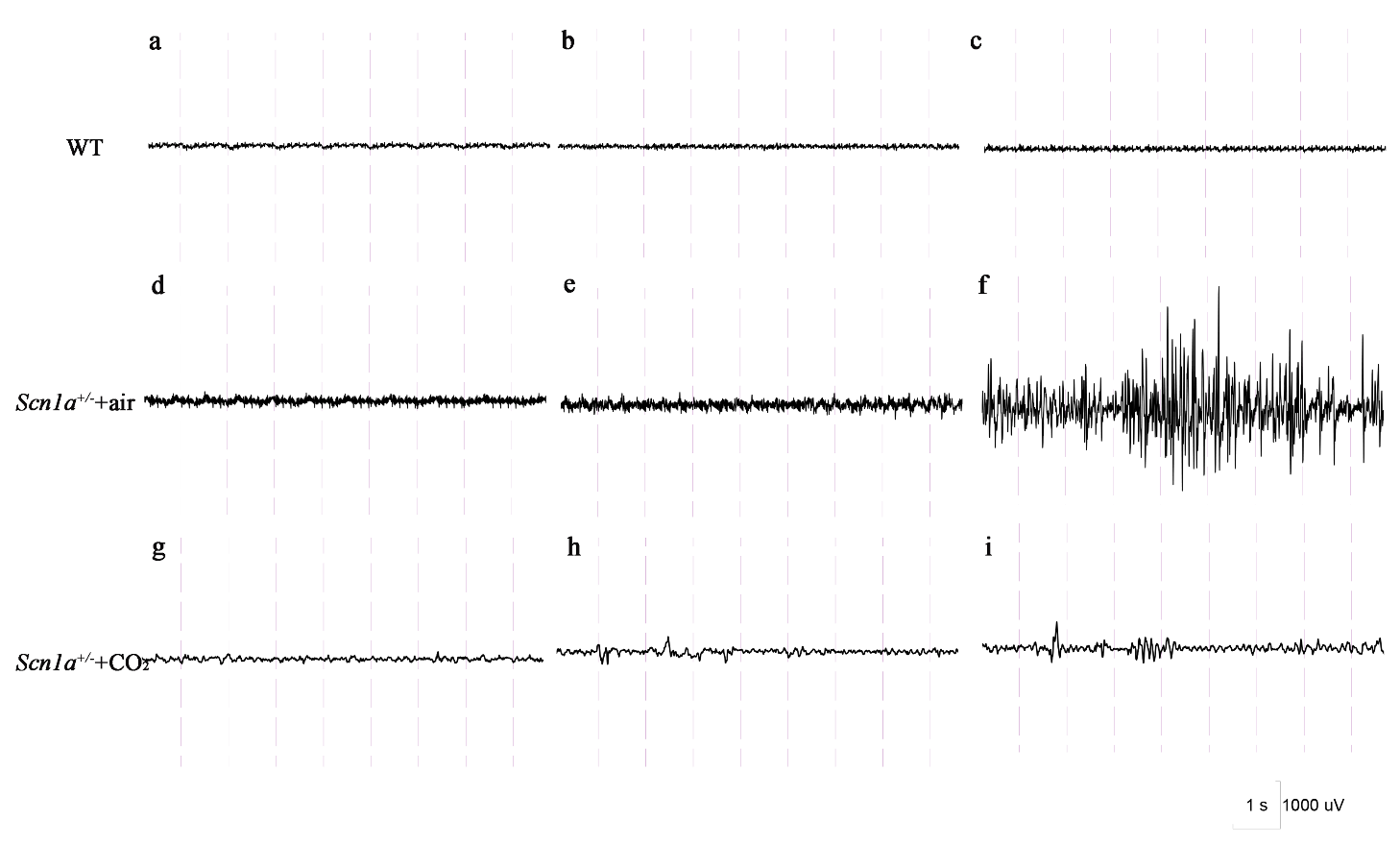


**Figure S2:** EEG results of mice. CO_2_ can reduce the EEG discharge of *Scn1a^+/-^* group. **Figures a-c** show the WT group, **Figures d-f** show *Scn1a^+/-^*+ air, and **Figures g-i** show *Scn1a^+/-^* + CO_2_ group. **Figures a, d and g** are normal body temperature, **Figures b, e and h** are gradually increasing body temperature, **Figures c and i** are 43℃, and **Figure f** is during a seizure.


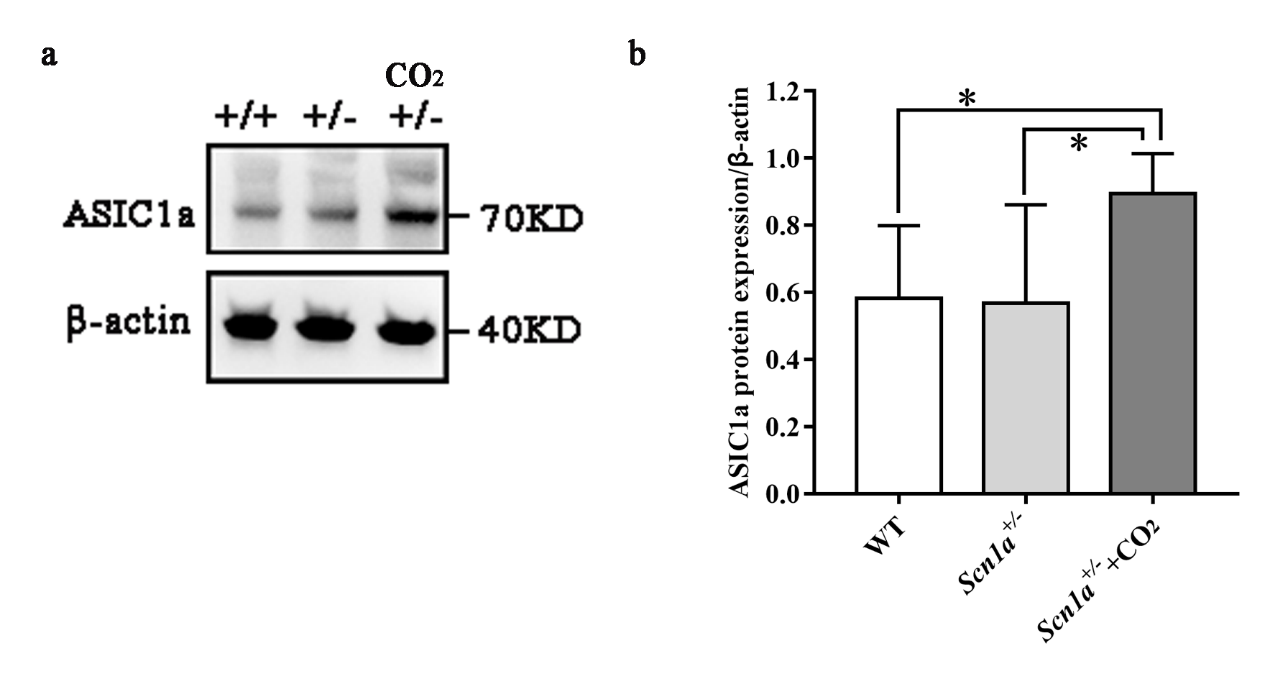


**Figure S3:** Increased expression of ASIC1a in the hippocampal region of *Scn1a^+/-^* + CO_2_ group. **Figure a** shows the WB results, +/+ is WT mice, and +/- is *Scn1a^+/-^* mice. **Figure b** shows the relative expression levels of ASIC1a protein/β-actin. * indicates *P*<0.05.
